# Exosomes derived from miR-26a-modified MSCs promote axonal regeneration via the PTEN/AKT/mTOR pathway following spinal cord injury

**DOI:** 10.1186/s13287-021-02282-0

**Published:** 2021-04-05

**Authors:** Yuyong Chen, Zhenming Tian, Lei He, Can Liu, Nangxiang Wang, Limin Rong, Bin Liu

**Affiliations:** 1 Department of Spine Surgery, The 3rd Affiliated Hospital of Sun Yat-sen University, Guangzhou, 510630 Guangdong China; 2 Guangdong Provincial Center for Quality Control of Minimally Invasive Spine Surgery, Guangzhou, 510630 Guangdong China; 3 Guangdong Provincial Center for Engineering and Technology Research of Minimally Invasive Spine Surgery, Guangzhou, 510630 Guangdong China

**Keywords:** Mesenchymal stem cells, Exosomes, Spinal cord injury, Axonal regeneration, miR-26a/PTEN axis

## Abstract

**Background:**

Exosomes derived from the bone marrow mesenchymal stem cell (MSC) have shown great potential in spinal cord injury (SCI) treatment. This research was designed to investigate the therapeutic effects of miR-26a-modified MSC-derived exosomes (Exos-26a) following SCI.

**Methods:**

Bioinformatics and data mining were performed to explore the role of miR-26a in SCI. Exosomes were isolated from miR-26a-modified MSC culture medium by ultracentrifugation. A series of experiments, including assessment of Basso, Beattie and Bresnahan scale, histological evaluation, motor-evoked potential recording, diffusion tensor imaging, and western blotting, were performed to determine the therapeutic influence and the underlying molecular mechanisms of Exos-26a in SCI rats.

**Results:**

Exos-26a was shown to promote axonal regeneration. Furthermore, we found that exosomes derived from miR-26a-modified MSC could improve neurogenesis and attenuate glial scarring through PTEN/AKT/mTOR signaling cascades.

**Conclusions:**

Exosomes derived from miR-26a-modified MSC could activate the PTEN-AKT-mTOR pathway to promote axonal regeneration and neurogenesis and attenuate glia scarring in SCI and thus present great potential for SCI treatment.

**Graphical abstract:**

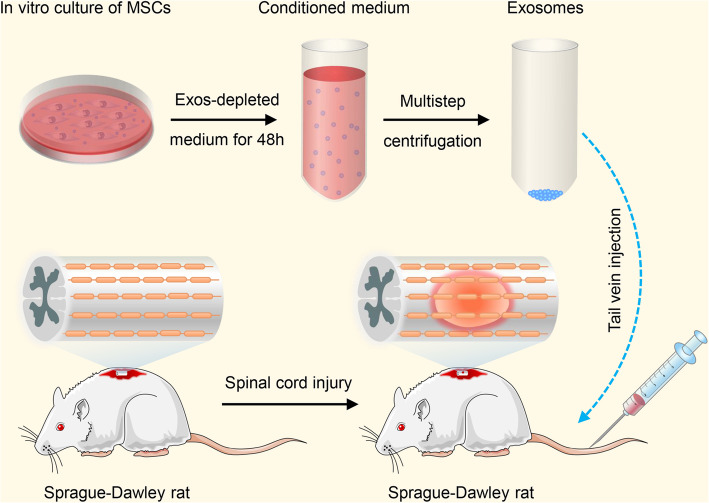

**Supplementary Information:**

The online version contains supplementary material available at 10.1186/s13287-021-02282-0.

## Introduction

Spinal cord injury is one of the devastating diseases that causes impaired neurological function and deterioration in the quality of life, has high morbidity and mortality, and imposes a heavy economic and social burden [1–4]. Although numerous therapies including drugs, physical therapy, hyperbaric oxygen therapy, and surgical interventions have been used clinically, none has been proven satisfactory due to the complex pathologic conditions [5]. The widespread cell death and severe inflammation caused by initial and secondary damage create a harsh environment for axonal regeneration [6–8]. Therefore, a novel treatment for SCI is urgently needed.

Mesenchymal stem cells have attracted more and more interest for SCI therapy with the development of cell transplantation. MSCs were proven to regulate immune response, reduce glial scarring, promote angiogenesis, direct neural transdifferentiation, and promote neurite remodeling [9–12]. However, most preclinical studies have reported a low survival rate of MSCs in the spinal cord after local grafting due to immune rejection [13–17]. Adverse effects of stem cell transplantation, including tumor formation and cell dedifferentiation, also limit the clinical application and development [18–20]. Further studies have shown that stem cell-induced neurological recovery relies more on intercellular communication processes than graft cell regeneration. Therefore, exosomes, the most prominent components of paracrine secretion, are considered a better choice for SCI treatment.

Exosomes are nanoliposomes with a diameter of 50–100 nm. Initially, the early sorted endosomes (ESE) are formed by endocytosis and the inward germination of the cell membrane, with extracellular components and cell surface proteins [21]. Then, the ESE is matured by Golgi, endoplasmic reticulum (ER), and mitochondria. ESEs produce multivesicular bodies (MVBs) and late sorting endosome. Partial MVBs are transported to lysosomes with or without autophagosomes for degradation. Others release their vesicles as exosomes. Exosomes were found to deliver their contents to target cell by receptor-ligand binding, direct membrane fusion, and endocytosis [22]. They mediate intercellular communication by transporting miRNAs, proteins, cytokines, mRNAs, etc. [23–25].

Exosome treatment shows therapeutic effect similar to direct transplantation of MSCs without inducing multiple adverse effects. The combined functional complexity of its contents provides the therapeutic efficacies of MSC exosomes [26]. To date, MSC exosomes have been reported to promote spinal cord injury recovery [27], improve graft-versus-host disease (GVHD) [28], reduce pulmonary hypertension [29], promote hepatic recovery [30], reduce myocardial ischemia/refusion injury [31], inhibit limb ischemia [32], and ameliorate wound healing [33].

miRNAs in exosome have been proven to play a crucial role in influencing target cells, gene expression, and signaling pathways [34–38]. miR-26 family is highly conserved, including miR-26a, miR-26b, miR-1297, and miR-4465. MiR-26a mature length is of 22 nucleotides and 7 nucleotides comprise about seed region, which is the key region for binding to the target mRNA. MiR-26a inhibits the expression of the protein encoded by the target gene through the imperfect sequence complementary binding to the target mRNAs [39]. Plenty of publications reported that miR-26a plays an important role in various diseases, such as regulating cell proliferation, apoptosis, angiogenesis in cancer cells, myocardial infarction, osteogenesis differentiation, bone regeneration, etc. [40–43]. In the central nervous system, miR-26a is highly expressed to regulate cell-cycle progression and neural progenitor differentiation [44, 45]. A previous study showed that miR-26a expression is decreased after SCI [46]. Additionally, increased miR-26a could enhance axonal outgrowth in hippocampal neurons and axonal regeneration in the peripheral nervous system [47]. While the downregulation of miR-26a leads to impaired axonal regeneration by suppressing GSK3β or PTEN expression in peripheral sensory neurons [48]. These results suppose that miR-26a has a key role in the control of axonal regeneration and SCI recovery.

PTEN was considered to be a target site of miR-26a in the regulation of angiogenesis, tumorigenesis, and myocardial ischemia [41, 42, 49, 50]. The PTEN/AKT/mTOR signaling pathway is an important regulator of cell growth, proliferation, metabolism, and viability. It also plays a crucial role in promoting axonal regeneration for central nervous system recovery [51–53].

However, only a few studies have focused on the therapeutic effect of this pathway in SCI. Our study was aimed to explore the effect of MSC-derived exosomes in repairing the injured spinal cord through miR-26a by modulating the mTOR pathway.

## Materials and methods

### Reagents and chemicals

The green fluorescent dye used in this study (PKH67) was obtained from Sigma–Aldrich (St. Louis, USA). Dulbecco’s modified Eagle’s medium (DMEM), phosphate-buffered saline (PBS), fetal bovine serum (FBS), and Triton X-100 were obtained from the Gibco Invitrogen Corporation (Gibco, USA). Furthermore, protease inhibitor cocktail, phosphatase inhibitor cocktail, Lipofectamine 3000, electrochemiluminescence (ECL) reagent, and the bicinchoninic acid protein assay (BCA) kit were obtained from Thermo Fisher Scientific (Thermo, USA). A Total RNA Kit was purchased from Omega Bio-tek (Omega Bio-tek, USA). A Prime Script RT Reagent Kit and SYBR Green PCR master mix were obtained from Takara Bio Inc. (Takara, Japan), and RIPA lysis buffer, Hoechst 33342, and SDS-polyacrylamide gels were purchased from by Beyotime Biotechnology (Beyotime, China). Paraformaldehyde (PFA) and horse serum (HS) were obtained from Biosharp Biotechnology (Biosharp, China) and Guangzhou Ruite Biotechnology (Ruite, China), respectively. Osteogenic, chondrogenic, and adipogenic differentiation kits were obtained from Cyagen Biosciences Inc. (Cyagen, China). CD9, CD63, β-tubulin-3 (Tuj-1), and Flotillin-1 antibodies were purchased from Abcam (Cambridge, UK), while miR-26a mimics, the corresponding negative control, and a U6 primer were purchased from RiboBio Biotechnology (RiboBio, China). Rapamycin was obtained from Invitrogen (Carlsbad, CA), and SN50 was obtained from Santa Cruz Biotechnology (Santa Cruz, CA). Additionally, NF was obtained from Genetex Biotechnology (Genetex, USA). PTEN, AKT, PI3K, mTOR, p-mTOR, p-AKT, and p-PI3K antibodies were purchased from Cell Signaling Technology (CST, USA), and AMPK, p-AMPK, ULK1, p-ULK1, S6K, p-S6K, p62, IKB, p-IKB, p65, and p-p65 antibodies were obtained from Affinity Biosciences (Affinity, USA).

### Bioinformatics and data mining

The GSE19890 dataset was used to explore changes in miRNA expression in a SCI rat model. Differences in miRNA expression on the seventh day after SCI were compared between 5 sham group samples and 5 injury group samples. The limma package of the R program was performed to analyze differences in miRNA expression. miRNAs with the *p* value lower than 0.05 and |logFC| > 1 were defined as differentially expressed miRNAs and are shown in the heat map.

In order to predict miRNAs related to the recovery of the nervous system, a special literature mining method was used to extract the miRNAs that have been verified by experiments. miR-26a was considered to play a crucial role in SCI treatment. The potential target genes of miR-26a were identified using the TargetScan (http://www.targetscan.org) and TargetMiner (http://www.mybiosoftware.com) databases to determine the functions of miR-26a. Furthermore, enriched GO terms and KEGG pathways for the miRNA target genes were determined by DAVID v6.7 (https://david.ncifcrf.gov).

### Isolation and characterization of bone marrow mesenchymal stem cells

Rat BMSC isolation and phenotype characterization were performed as described in the previous research [54]. Rat BMSC was obtained from the femur of 14-day-old SD rat. BMSC was cultured at 37 °C and 5% CO_2_ in a cell culture incubator. The culture medium was DMEM with 10% FBS. The medium was changed after the first 24 to 48 h, after which changed every 3 days. The BMSC at passage 3–5 was prepared for the following experiments.

CD29, CD34, CD44, CD45, and CD90 expression in BMSCs was detected by flow cytometry (FACSCanto™, USA) for BMSC phenotype characterization. Adipogenic, chondrogenic, and osteogenic differentiation was performed to determine the differentiation abilities of the BMSC.

The following medium was used to determine the multipotential differentiation capabilities of rat BMSCs: (1) chondrogenic differentiation medium (high-glucose DMEM, 40 μg/ml proline, ITS +premix (6.25 μg/ml bovine insulin and 6.25 μg/ml transferrin), 5.33 μg/ml linoleic acid, 50 μg/ml ascorbic acid, 100 nM dexamethasone, 1.25 mg/ml bovine serum albumin, 10 ng/ml TGFβ3, 1 mM sodium pyruvate, 6.25 μg/ml selenous acid); (2) adipogenic differentiation medium (10 nM dexamethasone, 0.1 mmol/L 3-isobutyl-1-methylxanthine, 50 μg/ml indomethacin, 10 μg/mL insulin, high-glucose DMEM, 10% FBS); and (3) osteogenic differentiation medium (50 μg/ml ascorbic acid, 10 mM β-glycerophosphate, 10 nM dexamethasone, 10% FBS, high-glucose DMEM). The medium was renewed every 3 days. The adipocyte was stained with Oil Red O, chondrocytes were stained with Alcian Blue for chondrocyte in pellet culture, and the osteocyte was stained with Alizarin Red S on day 14.

### Isolation and identification of MSC-derived exosomes

For collection of conditioned medium, the BMSC was cultured in DMEM with 10% exosome-free FBS for 48 h. To exclude dead cells and debris, the collected medium was successively centrifuged at 300×*g* for 10 min, 2000×*g* for 20 min, and 10,000×*g* for 30 min. The supernatant medium was ultracentrifuged at 100,000×*g* for 1 h. Pellets were then washed and resuspended in PBS before being centrifuged at 100,000×*g* for 1 h. BCA was performed to detect the exosome protein concentration.

Subsequently, transmission electron microscopy (HITACHI H-7000FA, Japan) was used to observe the morphology of the exosome. The particle size analysis was determined with the Zetasizer Nano system (Malvern, UK). Additionally, the protein levels of CD9 (1:1000), CD63 (1:1000), Flotillin-1 (1:10000), and Calnexin (1:1000) were measured using western blotting.

### Overexpression and detection of miR-26a exosomes (Exos-26a)

The BMSC was transfected with mimics or the corresponding negative control of miR-26a with Lipofectamine 3000. Conditioned medium was collected to extract exosomes. The total RNA was extracted and cDNA was produced using the total RNA Kit and the Prime Script RT Reagent Kit. SYBR Green Master Mix was used to perform qRT-PCR. The expression level was normalized to that of an internal control (U6) and then calculated by the 2^-ΔΔCt^ method. The sequences of the miR-26a mimics were as follows: 5-UUCAAGUAAUCCAGGAUAGGCU-3 and 5-AGCCUAUCCUGGAUUACUUGAA-3.

### PC12 cell culture and treatment

PC12 cells were obtained from the Shanghai Institute of Cell Biology, Chinese Academy of Sciences (Shanghai, China). Native PC12 cell was cultured in DMEM with 10% HS and 5% FBS. PBS or exosomes were added to medium in different groups. The PC12 cell in the Exos group was incubated with normal exosomes (20 μg/ml) for 48 h. PC12 cells in the Exos-26a group were incubated with miR-26a-overexpressing exosomes (20 μg/ml) for 48 h. PC12 cells in the RAP group were incubated with both miR-26a-overexpressing exosomes (20 μg/ml) and rapamycin (100 nM) for 48 h. The PC12 cell in the NF-κB inhibitor group was incubated with SN50 (100 μg/ml) for 48 h. After that, PC12 cells were prepared for immunofluorescence or western blotting.

### Evaluation of exosomes uptake

The exosome was stained with PKH67. To assess exosome uptake, the PC12 cell was incubated with PKH67-labeled exosomes (20 μg/ml) for 8 h. After that, the PC12 cell was fixed with 4% PFA. The nuclei were stained with Hoechst, and the fluorescence signal in PC12 cells was investigated.

### Immunofluorescence

The PC12 cell was fixed with 4% PFA for 0.5 h and blocked with 5% BSA for 1 h. The primary antibody against neurofilament (NF, 1:200) or glial fibrillary acidic protein (GFAP,1:200) was incubated overnight. An Alexa Fluor 555-labeled antibody was used to stain PC12 cells at room temperature. Hoechst 33342 staining was used to visualize the nuclei, and the laser confocal microscope (Zeiss LSM710, Germany) was used to observe fluorescent signal.

### Western blot analysis

The expressions of NF (1:1000), beta III tubulin 3 (Tuj-1,1:1000), GFAP(1:1000), PTEN(1:1000), AKT(1:1000), PI3K(1:1000), mTOR(1:1000), p-AKT(1:1000), p-PI3K(1:1000), p-mTOR(1:1000), AMPK(1:1000), p-AMPK(1:1000), ULK1(1:1000), p-ULK1(1:1000), S6K(1:1000), p-S6K(1:1000), p62(1:1000), IKB(1:1000), p-IKB(1:1000), p65(1:1000), and p-p65(1:1000) were determined using western blotting. RIPA lysis buffer was used to isolate protein with 1 mM phosphatase and protease inhibitor cocktail. Furthermore, SDS-PAGE and polyvinylidene fluoride membrane were used to separate the proteins. Primary antibody was used to incubate the blots overnight after blocking for 2 h. Secondary antibody was used to incubate the blots subsequently. The immunoreactive band was detected with ECL reagent by Tanon 5200 system (Tanon, China).

### Preparation of the experimental model

Male SD rats (6–8 weeks old) were used to establish the SCI model. After anesthesia with 2.5–3% isoflurane, a T9–T11 laminectomy was performed. After that, an aneurysm clip with the closing force of 75 g was used for compression at the T10 level for 30 s as previously described [55]. The muscles were then sutured immediately. The rats were distributed to several groups randomly (*n* = 6/group) and received injection of miR-26a exosomes (200 μg Exos-26a in 200 μL PBS), negative control exosomes (200 μg Exos in 200 μL PBS), or 200 μL PBS immediately following SCI via tail vein injection. The bladders of the rats were manually voided each day.

### Behavioral testing

The locomotor ability was investigated using Basso, Beattie and Bresnahan (BBB) scale. All animals were observed 1, 4, 7, 14, 21, and 28 days postinjury. Each rat was evaluated by 2 independent experimenters blinded to the treatment groups.

### Diffusion tensor imaging (DTI)

SD rats were placed in a scanner in the supine position after being anesthetized. All experiments were conducted on a 3.0-Tesla MR scanner (Discovery 750, General Electric, USA) with a dedicated animal coil (WK601-1085, Magtron Inc., China). Conventional DTI scans were then performed. Subsequently, all data were transferred to the General Electric AW 4.6 workstation, and the following parameters were used: *b* value of 800 s mm − 2, TE of 60 ms, TR of 3000 ms, 98 × 48 matrix, FOV 8 × 4, slice thickness of 2 mm, and 17 diffusion gradient directions.

### Motor-evoked potentials (MEPs)

The SCI rats were analyzed with MEPs to evaluate functional recovery 4 weeks postinjury [56, 57]. Rats were anesthetized with 2.5–3% isoflurane. The injured spinal cord was then exposed, a stimulation electrode was used, and recording and reference electrodes were inserted into the hind limb. A single square wave stimulus was subsequently applied, and the peak-to-peak amplitudes of MEPs were determined to assess nerve conduction in the hind limbs of the rats.

### Histological and immunofluorescence analysis

Rats were sacrificed and perfused with 4% PFA at 4 weeks postinjury. The T9–T11 segments were gently collected for histological evaluation. After fixation overnight in 4% PFA, paraffin, and hematoxylin eosin (HE) were used to embed and stain the spinal cord tissues.

A freezing microtome was used to cut the spinal cord tissues for immunofluorescence. The frozen slices were blocked with 10% BSA, and primary antibodies against NF, Tuj-1, and GFAP were used to incubate overnight, followed by secondary antibody for 1 h. The slice was stained with Hoechst. A panoramic viewer (Panoramic DESK, 3D HISTECH, Magyarorszag) was used to obtain fluorescence images.

### Statistical analysis

All data are shown as the mean ± S.D. Differences between multiple groups were analyzed using one-way ANOVA or *t* tests. Differences of a *P* value < 0.05. were considered statistically significant.

## Result

### Identification of miR-26a as a differentially expressed miRNA and its predicted target genes in a public dataset

**Fig. 1:**
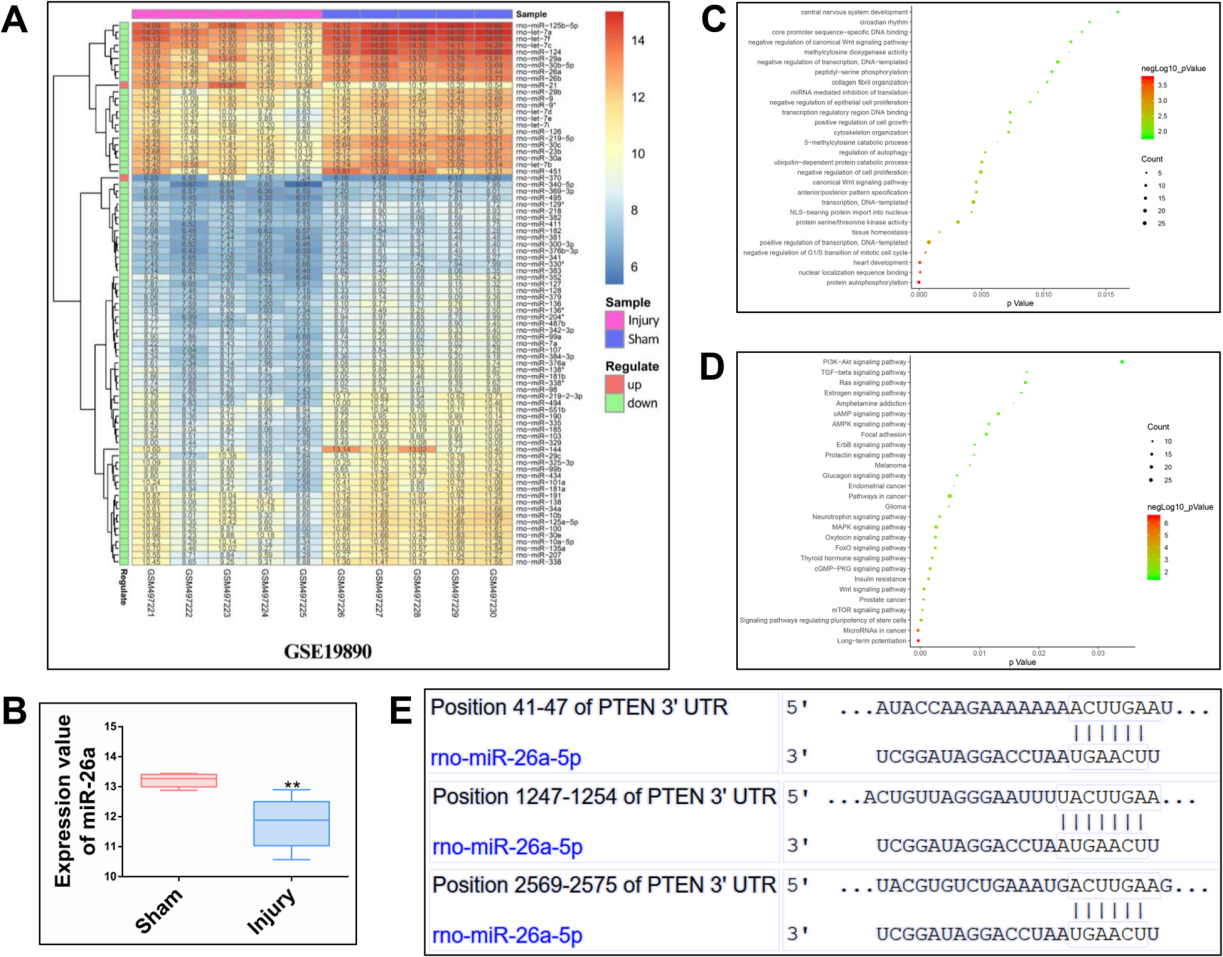
Identification of miR-26a as a differentially expressed miRNA and its predicted target genes. **a** Heat map of differentially expressed miRNAs based on the GSE19890 dataset. **b** miR-26a expression level following spinal cord injury. **c**, **d** Enriched GO terms and KEGG pathways for miR-26a target genes. **e** miR-26a target site in the 3′-UTR of PTEN. **P < 0.01 compared with the sham group by t test. *n* = 5 for each group

A total of 82 differentially expressed miRNAs were determined in the GSE19890 dataset. miR-26a expression was found to be downregulated as shown in Fig. 1a and b. By mining the scientific literature, miR-26a was found to be related to SCI recovery. Enriched GO terms and KEGG pathways for the miR-26a target genes were shown in Fig. 1c and d. The target genes of miR-26a are involved in multiple biological processes, such as central nervous development, regulation of cell migration, cell proliferation, and autophagy. The related signaling pathways include the mTOR signaling pathway, focal adhesion, and Foxo signaling pathway. According to the data, the PTEN and mTOR pathway were considered to be the close targets of miR-26a (Fig. 1e).

### Characterization of BMSC and BMSC-derived exosomes

**Fig. 2:**
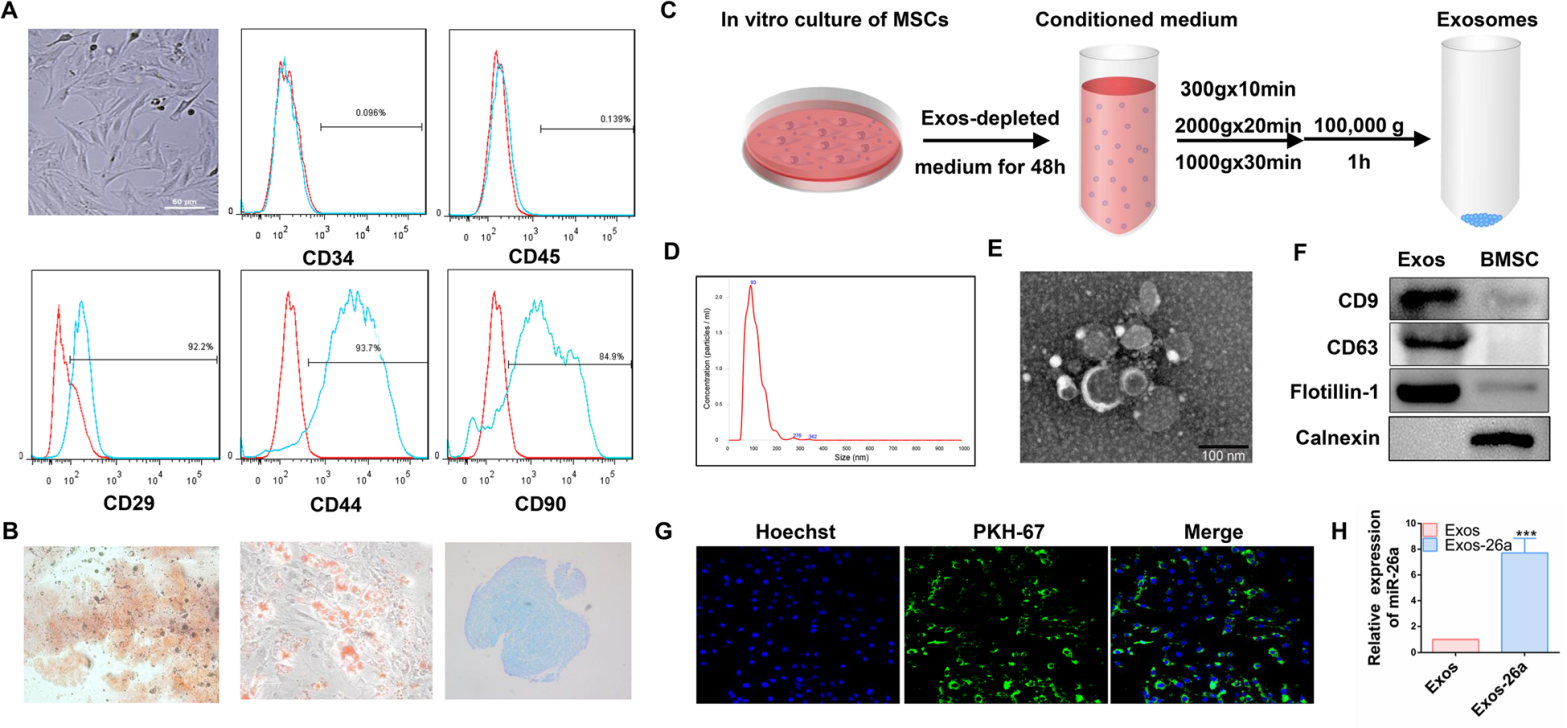
Characterization and overexpression of exosomes. **a**, **b** Isolation and characterization of MSC. **c** Schematic diagram of exosome isolation. **d** Nano measurements of exosomes. **e** Representative transmission electron micrographs of exosomes showing cup-shaped morphology. **f** Verification of expression of surface markers, including CD9, CD63, Flotillin-1, and non-exosomal marker Calnexin on exosomes by western blot analysis. **g** The uptake of green fluorescent dye-expressing exosomes into PC12 cells. **h** miR-26a expression level in BMSC-derived exosomes following miR-26a mimic transfection (Exos-26a group). ***P < 0.001 compared with the Exos group by *t* test. *n* = 3 for each group

The BMSC was positive for CD90, CD29, and CD44, but negative for CD34 and CD45 by flow cytometry analysis (Fig. 2a). In addition, the Alizarin Red, Alcian Blue, and Oil Red O staining demonstrated multiple differentiation potential of BMSC (Fig. 2b).

Figure 2c shows a schematic diagram of exosome production. BMSC-conditioned medium was centrifuged with 200 mL and purified to 100–150 μg of exosomes. As shown in Fig. 2d, nanomeasurements indicated that the diameters of the exosome particles ranged from 50 to 100 nm. Exosomes exhibited a cup-shaped morphology in Fig. 2e. Furthermore, surface markers, including CD63, CD9, and Flotillin-1, were positive on exosomes and the non-exosomal marker Calnexin was negative by western blot analysis (Fig. 2f).

### Evaluation of exosome uptake and overexpression

As shown in Fig. 2g, exosomes accumulated inside PC12 cells, which demonstrated that a significant number of exosomes was taken up. Additionally, the miR-26a expression level in BMSC-derived exosomes in the Exos-26a group was significantly increased following miR-26a mimic transfection (Fig. 2h).

### Exos-26a promoted neurofilament generation in PC12 cell via the mTOR pathway

**Fig. 3:**
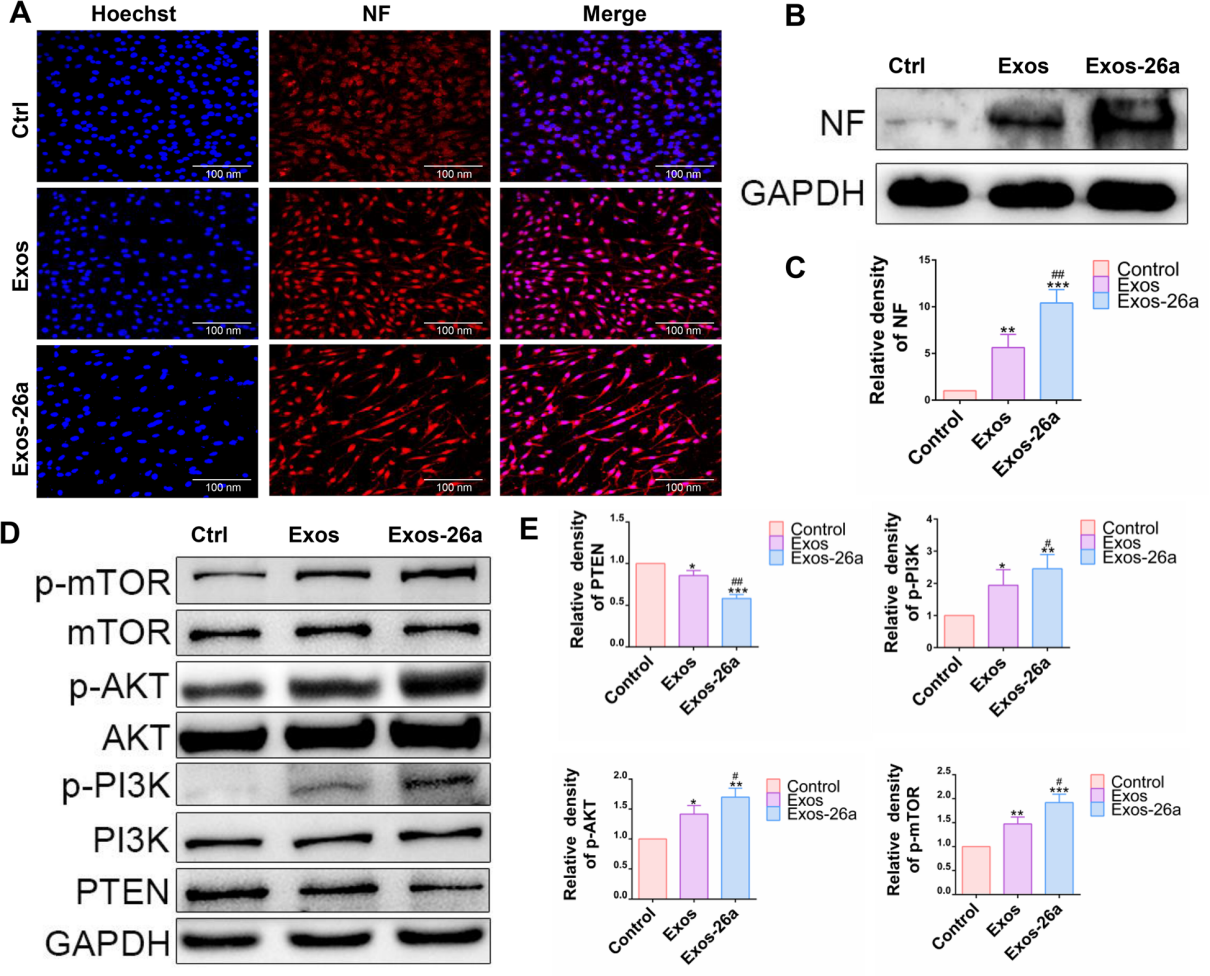
Exos-26a promoted neurofilament generation via the PTEN-AKT-mTOR pathway. **a** The ability of Exos-26a to generate neurofilament (red fluorescent dye) in PC12 cells. **b**, **c** Representative images of western blots for analysis of the expression level of NF and semiquantification of the data. **d**, **e** Representative images of western blots for analysis of the expression of PETN-AKT-mTOR pathway-related proteins and semiquantification of the data. The data are normalized to the control group. ^*^P < 0.05, ^**^P < 0.01, and ***P < 0.001 compared with the control group by *t* test or ANOVA test. ^#^*P* < 0.05 and ^##^*P* < 0.01 compared with the Exos group by *t* test. *n* = 3 for each group

The effect of Exos-26a on neurofilament generation in PC12 cells was evaluated. Figure 3a shows significant NF regeneration in the Exos-26a group but less NF regeneration in the other two groups. The expression level of NF was analyzed in Fig. 3b and c. Furthermore, analysis of proteins related to the PETN-AKT-mTOR pathway showed that Exos-26a targeted PTEN and downregulated its expression. As a result, the phosphorylation of PI3K, AKT, and mTOR protein was increased to promote NF generation and nerve regeneration (Fig. 3d, e).

### Exos-26a treatment promoted functional recovery following SCI

**Fig. 4:**
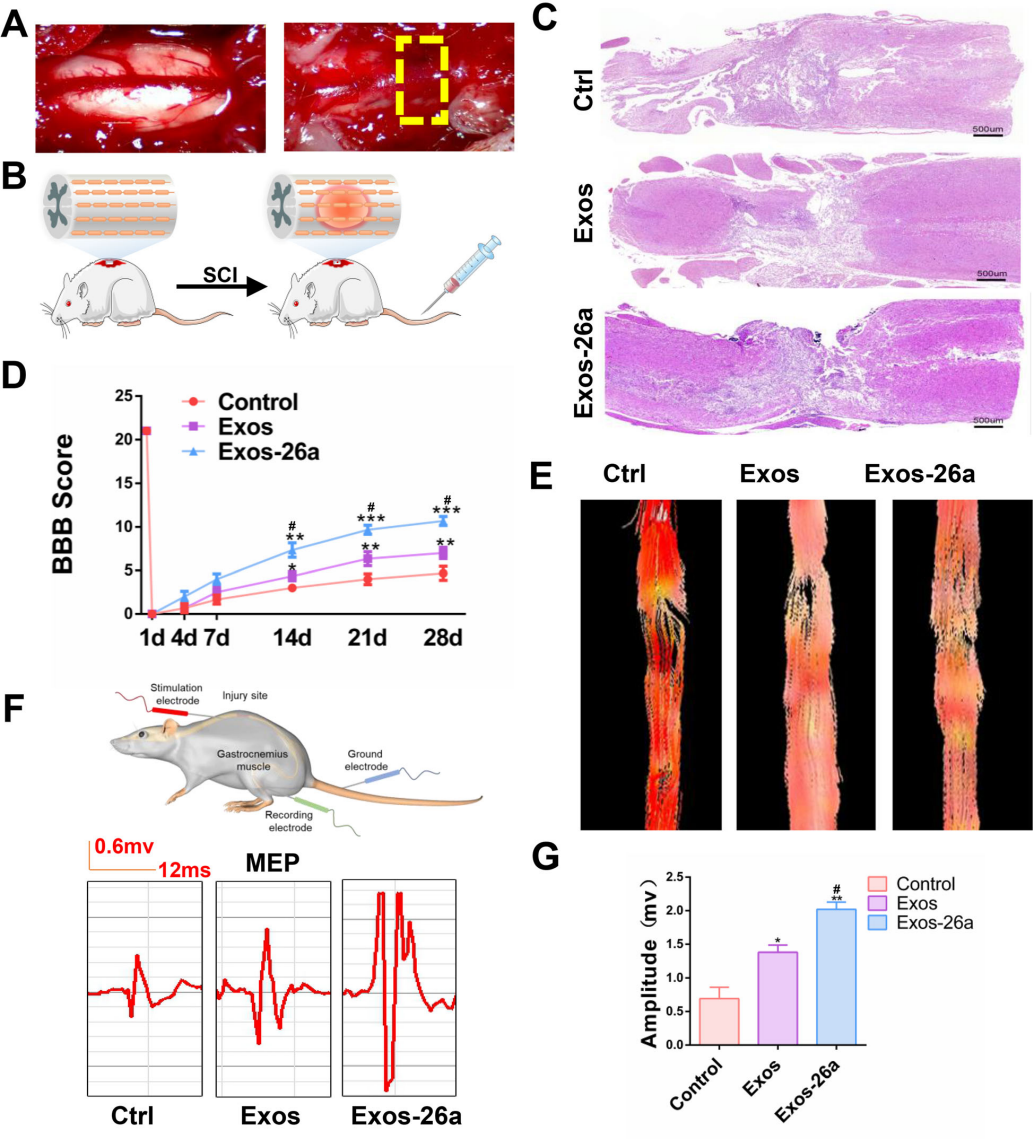
Exos-26a treatment promoted functional behavioral recovery following SCI. **a** Before and after compression of the spinal cord. **b** Schematic diagram of the method used to establish a spinal cord injury model and exosome treatment. **c** Hematoxylin and eosin staining of the PBS group, exosome negative control (Exos) group, and miR-26a-overexpressing exosome (Exos-26a) group on day 28 postinjury. **d** BBB scores of the three experimental groups. **e** DTI images of all groups on day 28 postinjury. **f**, **g** MEP amplitudes of the three groups on day 28 postinjury and quantification. ^*^P < 0.05, ^**^P < 0.01, and ***P < 0.001 compared with the control group by *t t*est or ANOVA. ^#^*P* < 0.05 compared with the Exos group by *t* test. *n* = 6 for each group

To investigate whether miR-26a-overexpressing exosomes could lead to better functional recovery following SCI, we first evaluated the functional recovery of rats in different groups using the BBB scale. The injured spinal cord before and after compression were presented in Fig. 4a. Schematic of the method used to establish the spinal cord injury model and exosome or PBS treatment was showed in Fig. 4b. Rats in the Exos group presented less cavity formation in the injured area and better functional recovery than rats in the other groups (Fig. 4c, d). Furthermore, BBB scores in the Exos-26a group increased significantly compared with the other groups. The results revealed smaller cavity formation in the Exos-26a group than the other 2 groups. Assessment of DTI images of rats also verified the previous results (Fig. 4e). To further investigate the effects of Exos-26a, MEP analysis was performed, as shown in Fig. 4f, g. In the Exos-26a group, amplitudes of MEP were improved better than those in the other groups. To sum up, these results indicated that treatment with Exos-26a could promote better functional recovery than control exosomes or vehicle in rats with SCI.

### Exos-26a treatment facilitated axonal regeneration and inhibited reactive astrogliosis after SCI

**Fig. 5:**
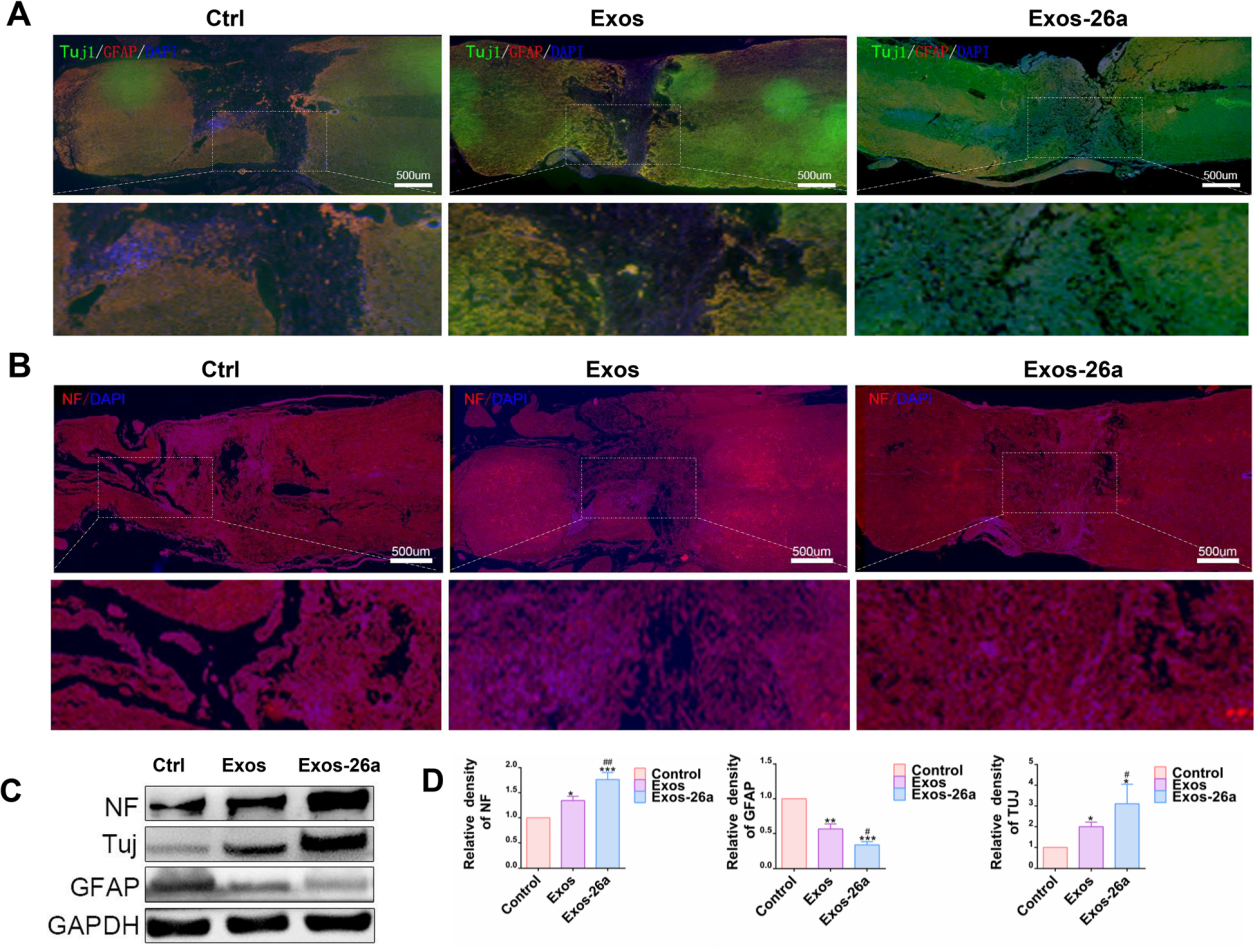
Exos-26a facilitated neuronal regeneration and inhibited reactive astrogliosis. **a** Representative immunostaining images of NF200 (red) in the injured areas of the spinal cord on day 28 postinjury. **b** Representative immunostaining images of Tuj-1 (green) and GFAP (red) in the injured areas of the spinal cord. **c** Western blot analysis of NF, Tuj-1, and GFAP in lesioned spinal cord segments and semiquantification of the data. ^*^P < 0.05, ^**^P < 0.01, and ***P < 0.001 compared with the control group by *t* test or ANOVA. ^#^*P* < 0.05 and ^##^*P* < 0.01 compared with the Exos group by *t* test. *n* = 3 for each group

To determine the neuropathological mechanism of previous functional improvement, we assessed the statuses of axons, neurons, and astrocytes, which play a vital role in SCI repair. NF and Tuj1 were used as markers to assess neuroregeneration after SCI. Increased GFAP expression is an indicator of astrocytic hypertrophy and has adverse effects in SCI. Immunostaining analysis of NF, Tuj-1, and GFAP revealed that the NF expression in the injured areas was upregulated in Exos-26a group at 4 weeks postinjury (Fig. 5a). Furthermore, a significant number of neurons and an occasional glial scar were observed in the lesion area and adjacent to the epicenter in the Exos-26a group but not in the control and Exos groups, as shown in Fig. 5b. NF, Tuj-1, and GFAP expression in lesioned spinal cord segments showed similar results (Fig. 5c, d) and indicated Exos-26a promoted neuronal and axonal regeneration and inhibited astrocyte inflammation, leading to better functional recovery following SCI.

### Exos-26a promoted functional recovery via the mTOR pathway

**Fig. 6:**
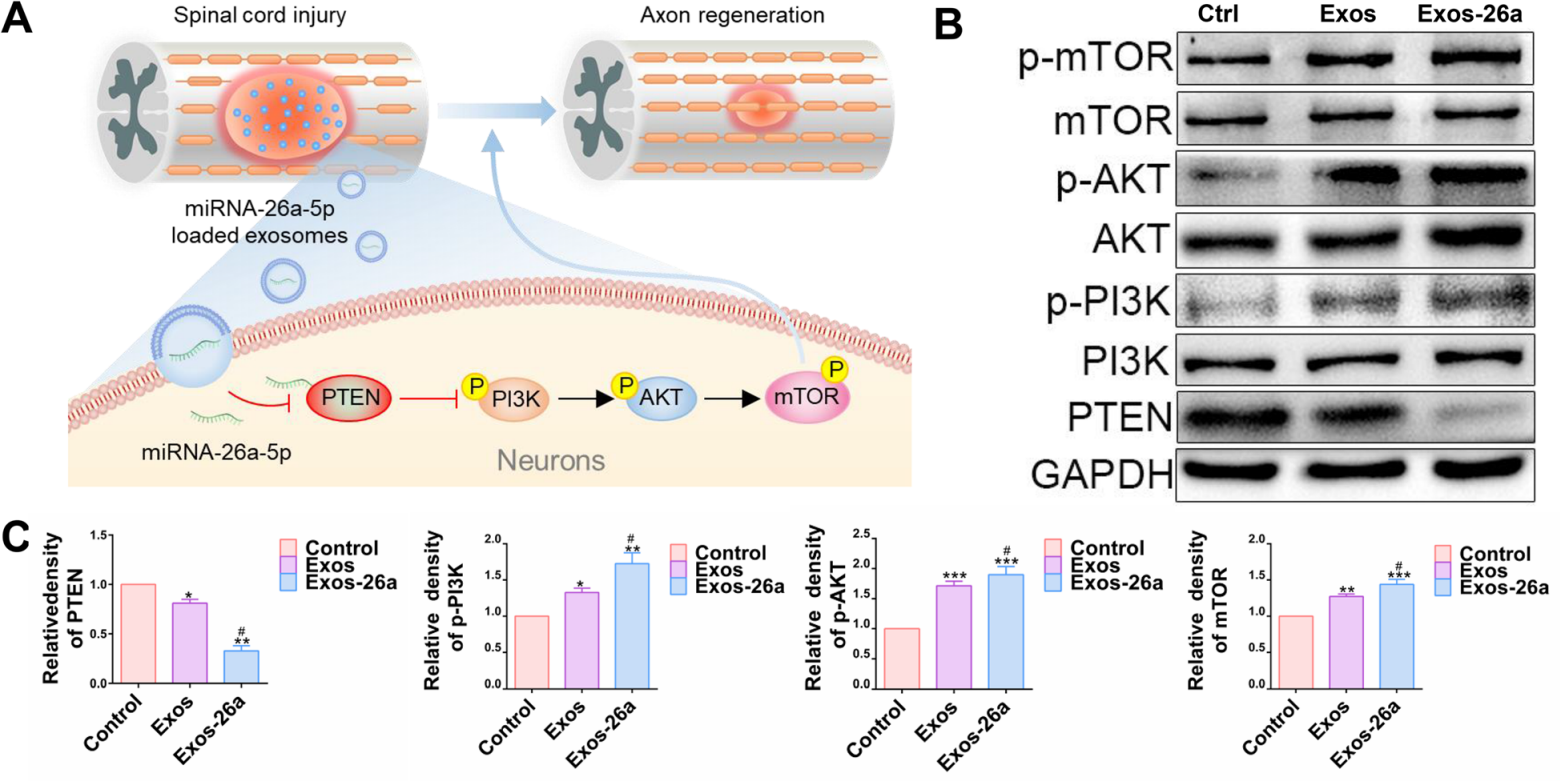
Exos-26a promoted spinal cord injury repair via the PTEN-AKT-mTOR pathway. **a** Diagram of the mechanism underlying the effect of Exos-26a via the PTEN-AKT-mTOR pathway. **b**, **c** Representative images of western blots used to analyze the expression of PETN-AKT-mTOR pathway-related proteins and semiquantification of the data. ^*^P < 0.05, ^**^P < 0.01, and ***P < 0.001 compared with the control group by *t* test or ANOVA. ^#^*P* < 0.05 compared with the Exos group by *t* test. *n* = 3 for each group

In our research, Exos-26a was demonstrated to provide better therapeutic effects for SCI. To further explore the possible mechanism, analysis of proteins related to the mTOR pathway was performed. Schematic of the mechanism underlying the effect of Exos-26a via the mTOR pathway was showed in Fig. 6a. The results showed that Exos-26a downregulated the expression of PTEN and upregulated the phosphorylation of the AKT, PI3K, and mTOR proteins (Fig. 6b, c). In conclusion, miR-26a-overexpressing exosomes could target PTEN and regulate the mTOR pathway to promote neuronal and axonal regeneration for SCI repair.

### Exos-26a enhanced mTOR activation, attenuated excessive autophagy, and increased axonal generation

To provide a more comprehensive assessment of the investigated signaling pathway, autophagic activity was analyzed by measuring p62, AMPK, pAMPK, S6K, pS6K, ULK1, and pULK1 levels. Exos-26a decreased the phosphorylation of AMPK and ULK1 while increasing the expression of p-S6K and p62 in the SCI rat model in Supplemental Figure 1A and B. Furthermore, Exos-26a showed a similar effect in PC12 cells: Exos-26a enhanced mTOR activation, attenuated excessive autophagy, and increased axonal generation, which could be reversed by rapamycin (Supplemental Figure 2A, B and C). In conclusion, miR-26a-overexpressing exosomes targeted and activated the mTOR pathway to attenuate excessive autophagy and promote axonal regeneration for SCI repair.

### Exos-26a exerted a similar effect as an NF-κB inhibitor in promoting axonal regeneration and inhibiting astrogliosis

As shown in Supplemental Figure 3, Exos-26 exhibited a similar effect as the NF-κB inhibitor in increasing the expression of NF and attenuating GFAP expression in vitro. The phosphorylation of IKB and p65 was also decreased in the Exos-26a group, which suggests that miR-26a inhibited the NF-κB pathway and had a similar effect as an NF-κB inhibitor in promoting axonal regeneration and inhibiting astrogliosis. Furthermore, the NF-κB signaling was also evaluated in the SCI rat model treated with exosomes on day 28 post injury (Supplemental Figure 4A and B). The phosphorylation of IKB and p65 were decreased in Exos-26a group. In summary, miR-26a was supposed to inhibit the NF-κB pathway and improve SCI recovery.

## Discussion

Functional recovery of SCI is usually less than ideal and there are only a few effective therapies in the clinic up to now [5]. Currently, treatments that can promote axonal regeneration and suppress astrocytic scarring and neuroinflammation are attracting the attention of researchers [58]. Stem cell therapy has shown considerable potential for SCI treatment in recent decades [59–61]. However, several studies have found that transplantation of MSCs provides few positive efficacies on SCI due to the blood-brain barrier and immunological rejection [62, 63]. Consequently, MSC-derived exosome, which has the ability to pass through the barrier and a low risk of immune rejection have become another potential treatment for SCI [64].

Exosomes contain many miRNAs that are involved in SCI pathogenesis and repair. Therefore, our study investigated the effects and underlying mechanism of miR-26a-overexpressing MSC-derived exosome in SCI therapy. Bioinformatics analysis and data mining revealed that miR-26a is differentially expressed after SCI. In this research, we verified that exosomes transplantation could improve functional recovery in a SCI rat model, which is consistent with some previous studies [65, 66]. Hematoxylin and eosin staining, assessment with the BBB scale, DTI, and MEP recording revealed more significant improvements in the miR-26a-overexpressing exosome group than the other groups. These miR-26a-overexpressing MSC-derived exosomes were shown to better facilitate axonal regeneration and prevent glial scarring after SCI than normal exosomes.

The possible mechanism of axonal regeneration induction by miR-26a exosomes was further researched in this study. miR-26 has a known role in tissue growth and neuronal development [67, 68]. A recent study showed that miR-26a induces angiogenesis via PI3K/AKT following cerebral infarction [69]. Furthermore, miR-26a activity can regulate neuronal morphogenesis and dendritic complexity through astrocyte-derived small extracellular vesicles [70]. Increased miR-26a-5p expression can enhance axonal outgrowth and regeneration in the nervous system [71, 72]. In our research, we verified that miR-26a-overexpressing exosomes had the ability to upregulate the NF and Tuj-1 expression, thereby increasing axonal regeneration.

PTEN was supposed to be a crucial negative regulator of the mTOR pathway that regulates axonal regrowth, neuronal survival, and functional recovery following CNS injury [73, 74]. Recent studies have reported that the deletion or inhibition of PTEN can activate the mTOR signaling, thereby exerting neuroprotective effects following nervous system injury [75–77]. The loss of PTEN has been revealed to increase neural stem cell differentiation and proliferation [78]. In our study, miR-26a-overexpressing exosome could downregulate the levels of PTEN and activate the mTOR pathway. Consequently, miR-26a-overexpressing exosomes exerted neuroprotective effects after SCI through the PTEN/AKT/mTOR pathways.

Autophagy is a crucial mechanism for bulk cytosolic degradation to maintain cellular homeostasis through the autophagosomal-lysosomal pathway. A recent study showed that activation of autophagy could be a novel strategy to treat neurodegenerative diseases [79, 80]. However, the differences in the functions of autophagy following SCI resulting from variations in the type of SCI, severity of SCI, or period of SCI are currently unclear. Tang et al. reported the trend in autophagy activation via LC3II expression analysis in hemisection spinal cord injury rat model [81]. Other researchers have also demonstrated that autophagy is enhanced in animals with contusion SCI [82]. Increased expression of p62 was investigated in a compression SCI model [83, 84]. Different types and severity of SCI could contribute to differences in the function of autophagy. In our study, a compression SCI model was established for in vivo investigation. The expression level of p62 was increased, which is consistent with previous literature. Furthermore, a previous research showed excessive autophagy is not beneficial for SCI repair. Björn Friedhelm Vahsen et al. proved that the inhibition of ULK1 can lead to mTOR activation, a decrease in autophagy, and an mTOR-mediated upregulation of proteins translation, therefore attenuating axonal degeneration [85]. Hong-Yu Zhang et al. also demonstrated that downregulation of excessive autophagy via the stimulation of mTOR pathway promoted functional recovery after SCI [86]. In this research, miR-26a-overexpressing exosomes exerted neuroprotective effects through PTEN/mTOR pathway activation and downregulation of excessive autophagy. However, autophagy plays a complicated role in SCI. More in-depth researches are needed to promote the clinical potential of miR-26a-overexpressing exosomes.

Mammalian NF-κB family contains 5 subunits, including Rel A (p65), c-Rel, Rel B, p50 (NF-κB1), and p52 (NF-κB2). NF-κB is considered to be closely related to the inflammatory response, cancer development, cellular proliferation, apoptosis, etc. [87]. NF-κB transcription factor is expressed in glial cells in the spinal cord, and its main function is to mediate a variety of mechanisms, such as immune response, injury response, and astrogliosis [88]. The astrocyte lineage spreads throughout the central nervous system and plays a crucial role in forming the blood-brain barrier and sending signals in the support and repair of neurons. Astrocytes play a key role in controlling the mechanism of SCI. Therefore, it is important to understand how to effectively target their activities to take advantage of their potential for repair and regeneration after SCI [89]. Multiple studies have demonstrated that downregulation of astroglial NF-κB signaling could improve functional recovery. The transgenic downregulation of astroglial NF-κB was found to inhibit astrogliosis and promote axonal regeneration following SCI [90, 91]. Furthermore, the inhibition of astroglial NF-κB could enhance oligodendrogenesis via regulation of the inflammatory response following SCI [92]. In our study, the expression of GFAP, a marker of the inflammatory response and astrocytic scarring, and NF-κB signaling were reduced by miR-26a exosomes. Therefore, miR-26a was supposed to be an important regulator in astrogliosis through NF-κB pathway for SCI therapy.

Multiple preclinical studies have verified the efficacy and safety of exosomes. In addition to SCI, exosomes are considered to play a critical role in cartilage regeneration, skin regeneration, liver repair, lung repair, etc. [93, 94]. Multiple clinical trials on exosomes have been conducted. To date, there are nearly 100 clinical studies registered online and the most widely used cell type is MSC. The other cell types includes NSC, EPC, and CPC (http://www.clinicaltrials.gov). MSCs are also the most commonly used type of stem cells in clinical trials, mainly because they have the advantage of being multipotent, can be easily isolated from adult tissues, and have greater ability to expand in vitro. Unlike embryonic stem cell or induced pluripotent stem cell, technically speaking, MSC is more suitable for our current regulatory framework, and there are fewer ethical disputes, which are considered to be a good source of exosomes therapy [26]. However, to our knowledge, results have been reported for only a few formal clinical trials on exosomes. To apply exosomes in clinical practice, more preclinical trials and clinical randomized controlled studies are needed.

## Conclusion

In summary, our study provides evidence that miR-26a-overexpressing exosomes have the potential to promote axonal regeneration following SCI. These processes could be regulated by PTEN, which is inhibited by miR-26a and subsequently activates the AKT/mTOR pathways. These findings indicate that administration of the combination of MSC-derived exosome and miRNAs could be a promising treatment approach for SCI.

## Supplementary Information

**Additional file 1: Supplementary Figure 1.** Evaluation of autophagic activity in a SCI rat model treated with exosomes. (a, b) Representative images of western blots used to determine the expression levels of AMPK, p-AMPK, S6K, p-S6K, ULK1, p-ULK1, and p62 and semiquantification of the data. ^*^P < 0.05, ^**^P < 0.01, and ***P < 0.001 compared with the control group by t test or ANOVA. ^#^*P* < 0.05 compared with the Exos group by t test. *n* = 3 for each group.

**Additional file 2: Supplementary Figure 2.** miR-26a-overexpressing exosomes inhibited autophagic activity and promoted axonal generation in PC12 cells. (a) The ability of Exos-26a to generate neurofilament (red fluorescent dye) in PC12 cells, which could be reversed by rapamycin. (b, c) Representative images of western blots used to determine the expression levels of NF, mTOR, p-mTOR, AMPK, p-AMPK, S6K, p-S6K, ULK1, p-ULK1, and p62 and semiquantification of the data. RAP indicates miR-26a exosome and rapamycin (100 nM) treatment for 48 h before lysis. ^*^P < 0.05, ^**^P < 0.01, ***P < 0.001 compared with the control group by t test or ANOVA. ^#^P < 0.05 and ^##^P < 0.01 compared with the RAP group by t test. *n* = 3 for each group.

**Additional file 3: Supplementary Figure 3.** miR-26a-overexpressing exosomes exert a similar effect as an NF-κB inhibitor in promoting axonal regeneration and inhibiting astrogliosis. (a) The ability of Exos-26a to generate neurofilament (red fluorescent dye) and inhibit glial fibrillary acidic protein (green fluorescent dye) in PC12 cells. (b, c) Representative images of western blots used to determine the expression levels of NF, GFAP, IKB, p-IKB, p65, and p-p65 and semiquantification of the data. ^*^P < 0.05, ^**^P < 0.01, and ***P < 0.001 compared with the control group by t test or ANOVA test. ^#^P < 0.05 and ^##^P < 0.01 compared with the NF-κB inhibitor group by t test. N.S., not significant. n = 3 for each group.

**Additional file 4: Supplementary Figure 4.** Evaluation of NF-κB signaling in a SCI rat model treated with exosomes. (a, b) Representative images of western blots used to determine the expression levels of IKB, p-IKB, p65, and p-p65 and semiquantification of the data. *P < 0.05 and **P < 0.01 compared with the control group by t test or ANOVA. #P < 0.05 compared with the Exos group by t test. n = 3 for each group.

## Data Availability

All data and materials are presented in the main paper.

## Abbreviations

SCI: Spinal cord injury
Exos: Exosomes
Exos-26a: miR-26a-overexpressing exosomes
BMSC: Bone marrow mesenchymal stem cell
BBB: Basso, Beattie and Bresnahan
DTI: Diffusion tensor imaging
MEP: Motor-evoked potentials
PBS: Phosphate-buffered saline
NF: Neurofilament
Tuj: Beta III tubulin
GFAP: Glial fibrillary acidic protein
GO: Gene ontology
KEGG: Kyoto Encyclopedia of Genes and Genomes
