# Supplementary figures and images for: Exosomes derived from miR-26a-modified MSCs promote axonal regeneration via the PTEN/AKT/mTOR pathway following spinal cord injury
Source: Stem Cell Res Ther. 2021 Apr 5;12:224. doi: 10.1186/s13287-021-02282-0 (PMC8022427; doi:10.1186/s13287-021-02282-0)

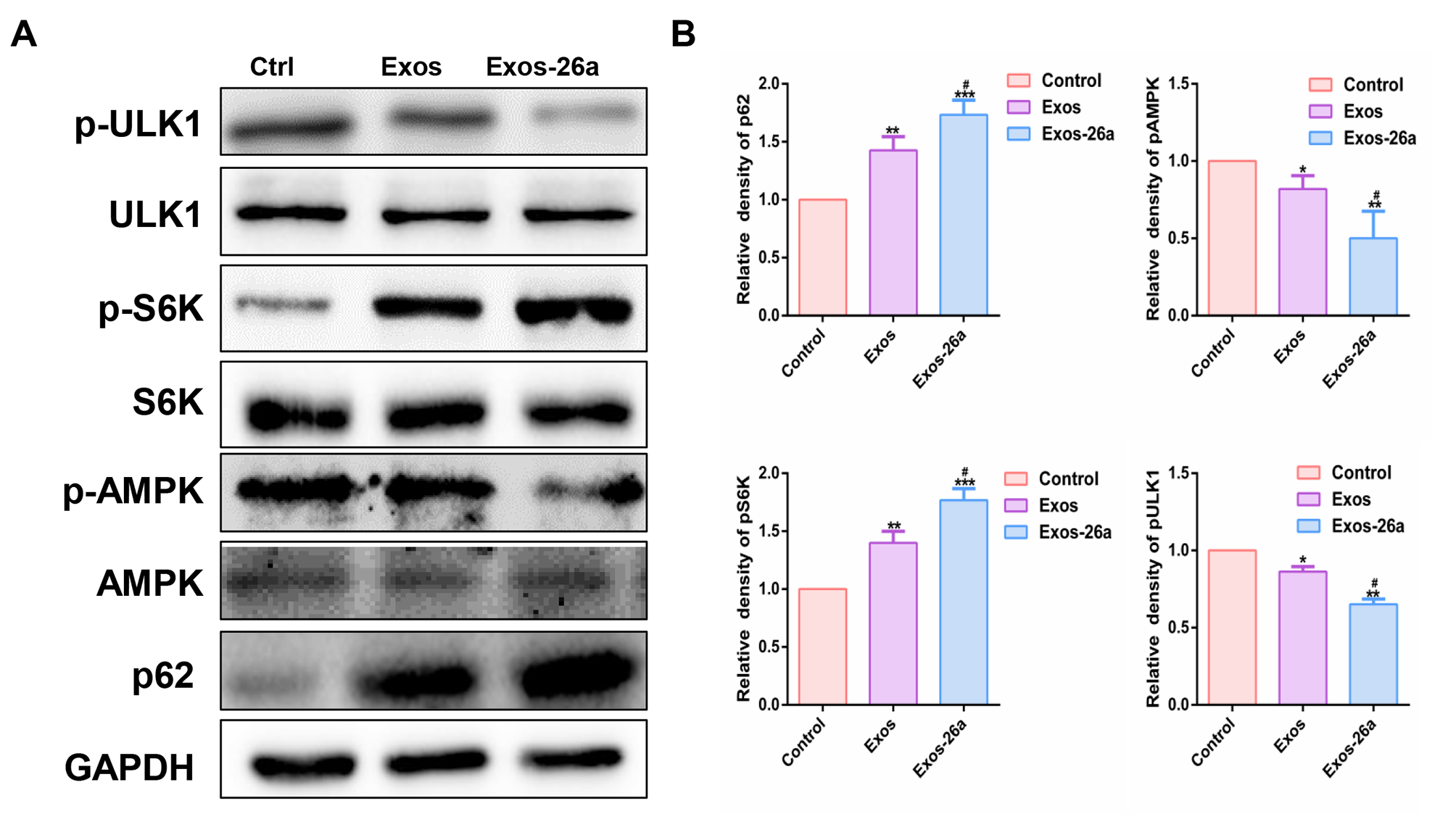

Supplement: Supplementary file 1 — Additional file 1: Supplementary Figure 1. Evaluation of autophagic activity in a SCI rat model treated with exosomes. (a, b) Representative images of western blots used to determine the expression levels of AMPK, p-AMPK, S6K, p-S6K, ULK1, p-ULK1, and p62 and semiquantification of the data. *P < 0.05, **P < 0.01, and ***P < 0.001 compared with the control group by t test or ANOVA. #P < 0.05 compared with the Exos group by t test. n = 3 for each group. [file 13287_2021_2282_MOESM1_ESM.tif]

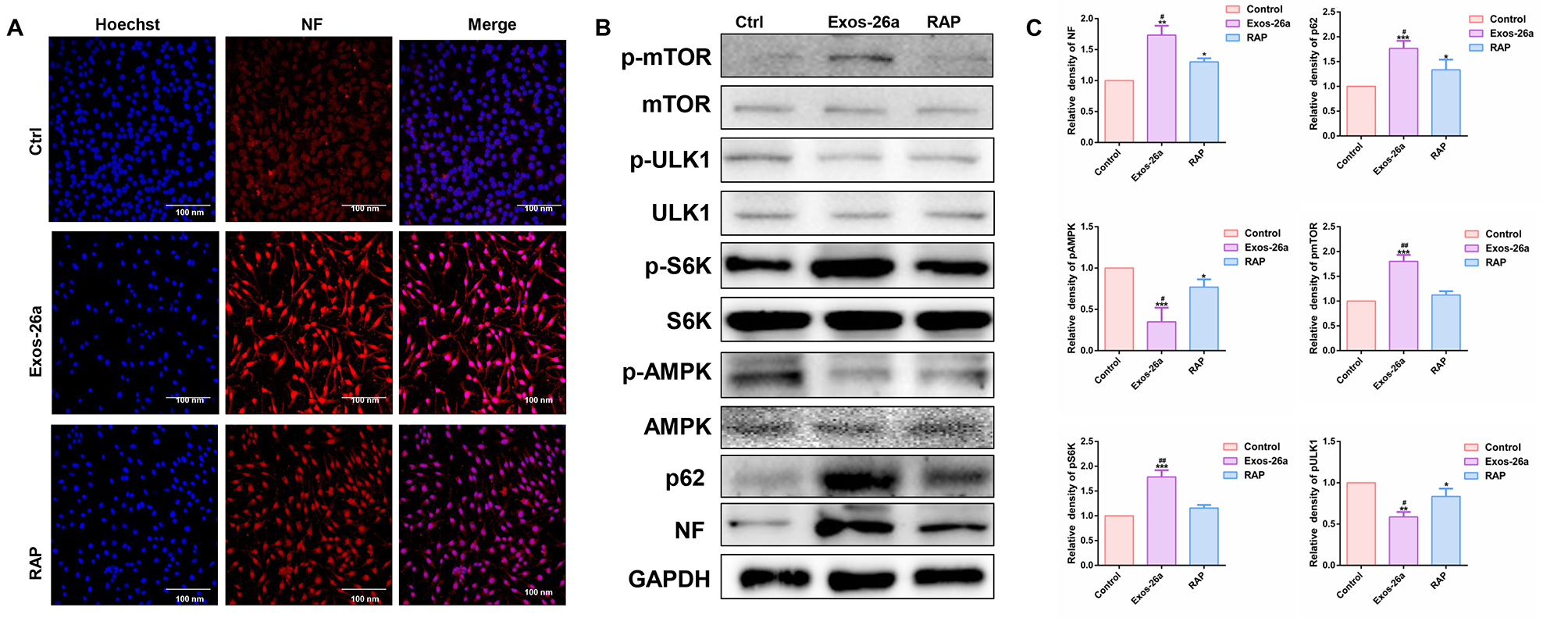

Supplement: Supplementary file 2 — Additional file 2: Supplementary Figure 2. miR-26a-overexpressing exosomes inhibited autophagic activity and promoted axonal generation in PC12 cells. (a) The ability of Exos-26a to generate neurofilament (red fluorescent dye) in PC12 cells, which could be reversed by rapamycin. (b, c) Representative images of western blots used to determine the expression levels of NF, mTOR, p-mTOR, AMPK, p-AMPK, S6K, p-S6K, ULK1, p-ULK1, and p62 and semiquantification of the data. RAP indicates miR-26a exosome and rapamycin (100 nM) treatment for 48 h before lysis. *P < 0.05, **P < 0.01, ***P < 0.001 compared with the control group by t test or ANOVA. #P < 0.05 and ##P < 0.01 compared with the RAP group by t test. n = 3 for each group. [file 13287_2021_2282_MOESM2_ESM.tif]

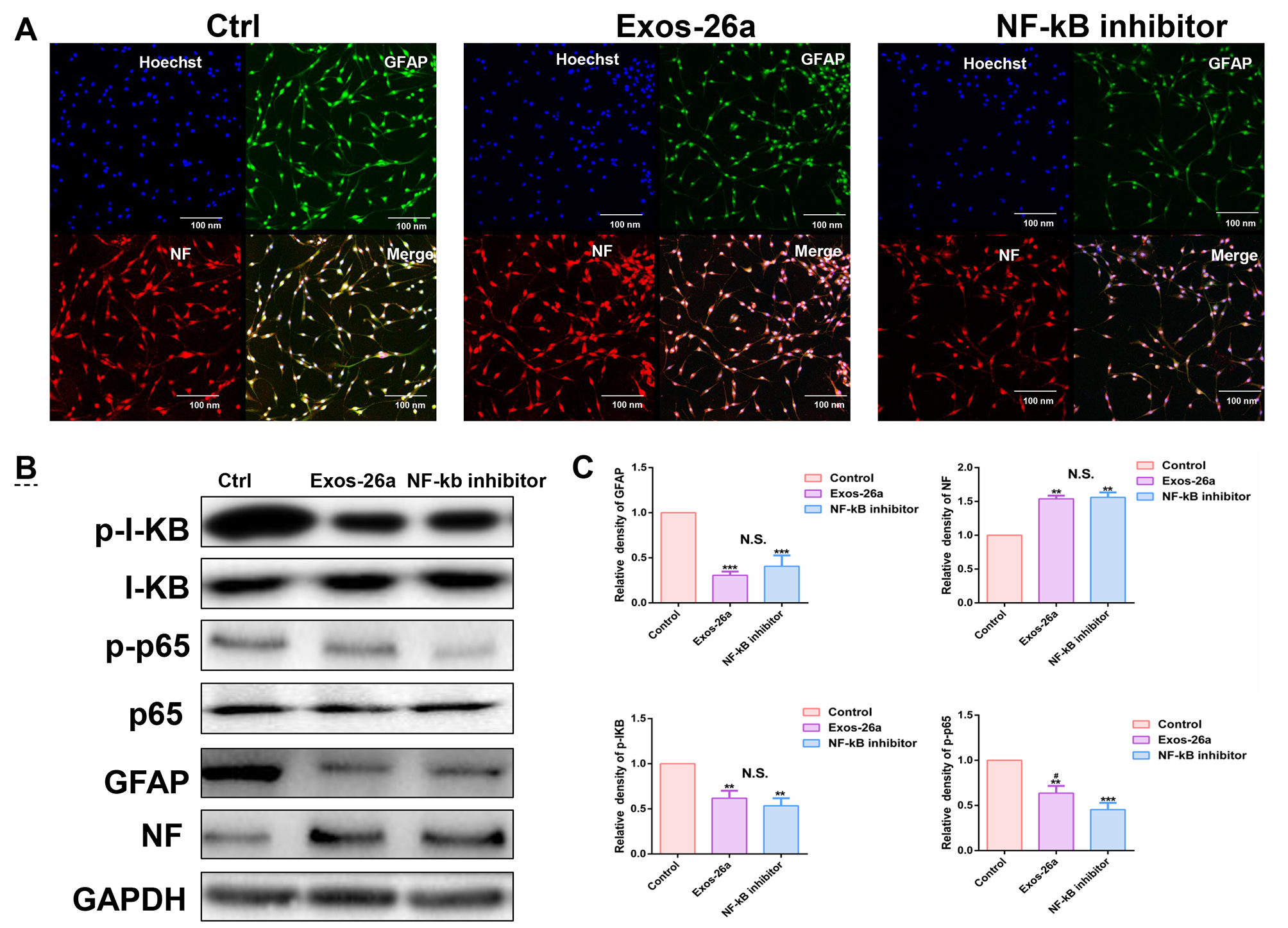

Supplement: Supplementary file 3 — Additional file 3: Supplementary Figure 3. miR-26a-overexpressing exosomes exert a similar effect as an NF-κB inhibitor in promoting axonal regeneration and inhibiting astrogliosis. (a) The ability of Exos-26a to generate neurofilament (red fluorescent dye) and inhibit glial fibrillary acidic protein (green fluorescent dye) in PC12 cells. (b, c) Representative images of western blots used to determine the expression levels of NF, GFAP, IKB, p-IKB, p65, and p-p65 and semiquantification of the data. *P < 0.05, **P < 0.01, and ***P < 0.001 compared with the control group by t test or ANOVA test. #P < 0.05 and ##P < 0.01 compared with the NF-κB inhibitor group by t test. N.S., not significant. n = 3 for each group. [file 13287_2021_2282_MOESM3_ESM.tif]

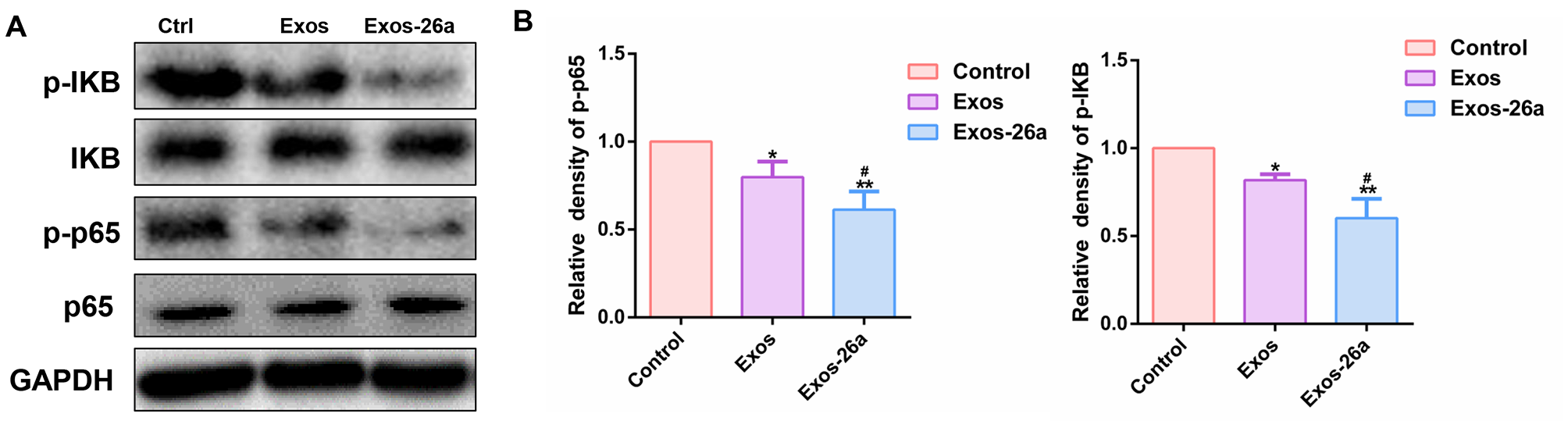

Supplement: Supplementary file 4 — Additional file 4: Supplementary Figure 4. Evaluation of NF-κB signaling in a SCI rat model treated with exosomes. (a, b) Representative images of western blots used to determine the expression levels of IKB, p-IKB, p65, and p-p65 and semiquantification of the data. *P < 0.05 and **P < 0.01 compared with the control group by t test or ANOVA. #P < 0.05 compared with the Exos group by t test. n = 3 for each group. [file 13287_2021_2282_MOESM4_ESM.tif]
